# How can sexual and reproductive health and rights be enhanced for young people with intellectual disability? – focus group interviews with staff in Sweden

**DOI:** 10.1186/s12978-020-00928-5

**Published:** 2020-06-03

**Authors:** Maria Wickström, Margareta Larsson, Berit Höglund

**Affiliations:** Department of Women’s and Children’s Health, Uppsala University, 751 85 Uppsala, Sweden

**Keywords:** Focus group interwievs, Human rights, Intellectual disability, Reproduction, Sexuality, Adolescent

## Abstract

**Background:**

Different types of staff support individuals with intellectual disability (ID) in their daily life, in schools, leisure activities and in special accommodations. This study aimed to gain a deeper understanding of experiences and perceptions regarding sexual and reproductive health and rights (SRHR) among staff.

**Methods:**

Data were collected in mid-Sweden in four focus groups with altogether 20 participants, 18 women and 2 men aged between 18 and 65 years. They had different professions and worked among youth and adults with ID aged 18–40 years in schools, accommodations and with leisure activities. Their working experience varied from 3 years to more than 20 years. Interviews were audio recorded, transcribed and analysed with content analysis.

**Results:**

The participants generally described positive attitudes towards sexuality for people with ID, both among themselves and in society. However, many situations such as ensuring privacy, balancing between waiting and acting, issues around contraception and reproduction were difficult to address and participants had hesitations about childbearing. They described different strategies such as showing respect, enhancing self-esteem and decision making ability and using interprofessional support to cope with frustrating situations. They lacked a clear mandate from managers as well as written guidelines and policies. They requested education and support from peers, supervisors and other professionals.

**Conclusion:**

Participants in the study were generally open-minded and accepting towards sexuality among young people with ID. They thought it was difficult to deal with reproduction/parenthood and felt unprepared and frustrated in certain situations. The participants requested a clear mandate from managers, organizational guidelines, more education and inter-professional support. We believe these findings can inform the development of policy and support the implementation of SRHR related guidelines to support staff working with young people with ID.

## Plain English summary

The Convention on the Rights of Persons with Disabilities states that individuals with intellectual disability (ID) should have the same rights to good sexual and reproductive health (SRH) as people without disabilities.

In Sweden, the Law on Support and Service for people with Certain Functional Impairments guarantees full participation in society, which means that young people with ID can live in special houses or apartments, supported by staff, or move into their own apartment. They have a right to help and support related to their own special needs and are thus often surrounded by different groups of professionals in their daily life. We wanted to get a deeper understanding of staff’s experiences and perceptions regarding sexual and reproductive health and rights (SRHR) for young people with ID.

Four focus groups interviews involving altogether 20 professionals, 18 women and 2 men, were performed and analysed with content analysis, which resulted in one overarching theme and several categories related to the theme.

The overarching theme: “Sexual and reproductive health and rights for young people with ID are gradually being accepted. Remaining uncertainty and frustration calls for improved policy and practice” describes positive attitudes towards sexuality for people with ID among the staff themselves and in society. However, many situations, especially in relation to reproduction and parenthood, were difficult to address and participants had hesitations about childbearing. They developed different strategies to cope with frustrating situations, but lacked a clear mandate from managers. They also requested education and support from peers, supervisors and professionals.

## Introduction

In 2006, The United Nations adopted the Convention on the Rights of Persons with Disabilities, which gives individuals with ID the same rights to good sexual and reproductive health (SRH) as people without disabilities. That includes the rights to retain fertility, to relationships, family and parenthood, to education on reproduction and family planning in an age- and development-appropriate manner [1].

In Sweden a Law of Care (1967:940), passed in 1968 became the starting point for elimination of segregated institutions for people with ID. The Law of Care stated that individuals with ID could live in integrated homes, have the right to education and access to activities at a day centre. This law was later adapted and replaced in 1994 by.

the Law on Support and Service for people with Certain Functional Impairments [2]. This law guarantees full participation in society, which means that young people with ID can move to houses or apartments where the staff assists them, or they can move into an apartment of their own. They have a right to apply for help and support related to their own special needs, e.g. daily care and support. The municipality is responsible for life-long support to this group. A person with ID has full legal control in terms of own rights and responsibilities but a trustee could be nominated to assist in economic and civil rights matters. The Nordic countries have similar laws on support and service for people with certain functional imparments [3].

Individuals with ID thus often have contact with different types of caregivers and professionals who support them in their daily life. A meta-synthesis described caregivers’ experiences and perceptions regarding sexual needs of people with ID. Five categories were found: *Fear and uncertainty, Impact of perceptions of sexuality, Same and different, Balancing the roles of protector and facilitator, Conditional sexuality: conditional support.* The findings suggest key issues for caregivers in relation to addressing the sexual needs of individuals with ID and highlight possible implications based on caregivers own views on their practice [4]. Another study described that it is complex and challenging to support individuals with ID regarding sexual relationships. Individuals with ID were described as lonely, disempowered and vulnerable to abuse. Moreover, the sex industry, internet and mobile phones were identified as new forms of risks [5]. Staff providers, compared to family carers, more often supported individuals with ID to have intimate and non-intimate relationships. Only 20% of the family carers considered that individuals with ID should be involved in the decision to engage in a relationship compared to 79% among the staff carers [6].

Women with ID seldom raise the question about contraception themselves [7, 8]. One study showed that half of the women who received contraception lacked basic knowledge and none had been given appropriate and adapted information [9]. Greenwood and Wilkinson suggest that clinicians who meet individuals with ID should, not only be aware of the risk of sexual abuse but also assess the individuals’ capability and desire to be pregnant and have a child [10]. One study showed, however, that parents and staff are less positive to parenthood than to sexual activities [11], and Swedish studies showed that midwives had unfavourable attitudes towards parenthood among people with ID [12, 13].

Rushbrooke et al. highlight the need for more research about caregivers’ experiences of providing support to people with ID concerning intimate relationships [4]. The aim of this study was to gain a deeper understanding of staff ‘s experiences and perceptions regarding sexual and reproductive health and rights (SRHR) related to individuals with ID.

## Method

### Design

Qualitative interview study with focus group interviews.

### Setting and sample

The study was carried out in mid-Sweden. As described earlier, individuals with mild to moderate ID in Sweden tend to participate in society to a great extent. They have the right to intimate relationships, sexual activities and childbearing and should receive appropriate support also in this domain of life. Individuals with severe ID more often live in institutions without the same mobility and possibilities to enter into relationships or form a family. Their needs and possibilities in relation to sexuality and reproduction are different and not in focus for this study.

We therefore targeted and purposefully recruited professionals who worked with youth and adults, 18–40 years, with mild to moderate intellectual disability. The participants in this study; 18 women and two men between 18 and 65 years worked in special accommodations, in special schools, in daily activities and had different types of professions. Their working experience varied from 3 years to more than 20 years. See Table 1.

**Table 1:** Characteristics of the informants

Socio-demographic data		Number (%)
**Age**	18–30 years	2 (10)
	31–40 years	4 (20)
	41–50 years	5 (25)
	51–65 years	9 (45)
	Total	20 (100)
**Gender**	Women	18 (90)
	Men	2 (10)
	Total	20 (100)
**Education**	College	6 (30)
	Advanced vocational education	1 (5)
	University	13 (65)
	Total	20 (100)
**Workplace**	Assistant at special accommodation	4 (20)
	Instructor at work	1 (5)
	Welfare officer	2 (10)
	Teacher	6 (30)
	Nurse	2 (10)
	Recreation instructor	2 (10)
	Teacher’s assistant	2 (10)
	Total	19 (95)
	Missing information	1 (5)
**Work experience**	3–5 years	1 (5)
	5–10 years	7 (35)
	11–20 years	6 (30)
	More than 20 years	6 (30)
	Total	20 (100)

### Data collection and procedure

To reach potential informants, a member of the study team contacted all managers at various workplaces in the region, first by telephone and then through e-mail, and provided written information about the study together with a written invitation letter intended for potential participants. The managers forwarded our request and the information letter to the staff. In a few workplaces, one member of the study team was invited to present the study and invited staff to participate. Those who reported an interest sent an e-mail to the study team, signed a written consent form and were assigned to one of four focus groups. The groups were composed with participants from different workplaces and with different professions in order to stimulate and broaden the discussion. The interviews were conducted in 2015. A topic guide with five topics; Sexual health, Sexual relations, Contraception and Pregnancy/Parenthood was designed based on previous studies [5, 14]; The initial question was: “What are your experiences/thoughts regarding people with ID and sexuality” and a card with the topic “Sexuality” was shown. The interview then continued with placing new cards on the table. One member of the study team acted as moderator and two other members, both midwives and researchers, alternated as facilitators. Before the sessions, the informants filled out a short questionnaire with socio-demographic data. Towards the end of each interview the moderator summarized what had been discussed in order for participants to add additional information or correct any misunderstandings. After four interviews data collection was stopped since very little new data emerged and saturation was deemed satisfactory. The interviews were audio recorded and transcribed verbatim.

### Analysis

Since knowledge in this area is limited, we used content analysis with a conventional or inductive approach [15, 16]. The texts were read several times to achieve a sense of the whole [16]. Meaning units were identified and condensed, while preserving the core. Condensed meaning units were labelled with a code, describing the content [17], see Table 2. The moderator worked with the coding process but repeatedly discussed and evaluated it with the co-authors. Together, all authors organised and sorted the codes into categories and sub-categories [16]. In the last step we identified a theme, describing the latent content of the data [17]. All categories and sub-categories are presented with illuminating quotes. The entire analytical process was done in Swedish, and a native English speaker translated the quotes presented in the manuscript.

**Table 2:** Examples of the analytical scheme

Meaning unit	Condensed meaning unit	Code	Sub-category	Category	Theme
I feel like I can’t explain to her. I look at her as soon as it comes to children near her that she expresses and she shows how much she would like to have her own and the way she talks about the children of others. I don’t know really; I’m just trying to be positive. I don’t know how to address it.	I feel that I can’t explain to her concerning children. I don’t know how to address her wish for a child.	Informant uncertain about how to address individuals who wish for a child.	Reproduction and parenthood are most difficult to address.	A complex issue to address for the staff.	Sexual and reproductive health and rights for young people with ID are gradually being accepted. Remaining uncertainty and frustration calls for improved policy and practice.

### Ethical considerations

All participants received verbal and written information before the interview and gave written consent. The moderator explained the structure of the focus group interview and that everyone could speak freely and that confidential data would be excluded from the analysis. The Regional Ethical Committee in Uppsala, Sweden approved the study (Dnr: 2014/414).

## Findings

The analysis resulted in three main categories, each with three to five sub-categories. We also formulated an overarching theme based on all categories and our interpretation of the findings (See Table 3).

**Table 3:** Overview of the findings

**Theme**	Sexual and reproductive health and rights for young people with ID are gradually being accepted. Remaining uncertainty and frustration calls for improved policy and practice.		
**Categories**	A society that begins to understand	A complex issue for the staff to address	Various strategies to work with SRHR
**Sub-categories**	A positive progress in society	Difficult and sometimes unmanageable work	Strategies to enhance good SRHR for individuals with ID
	The staff consider themselves open-minded in SRHR issues	Uncertainty based on level of knowledge and staffs’ options/obligations	Strategies to provide individuals with ID with knowledge about SRHR
	Society limits the individual	Relationship and contraception – a topic to address when it appears	Strategies to achieve a good working environment
	Deficiency in the structure of the organisation	Reproduction and parenthood are most difficult to address	
		Social norms and own preconceptions affect the staff’s work and approach	

*“Sexual and reproductive health and rights for young people with ID are gradually being accepted. Remaining uncertainty and frustration calls for improved policy and practice”* became the overarching theme*.* The staff noticed a positive progress in society, they considered themselves open-minded regarding SRHR and had developed strategies to work with SRHR-questions. “The uncertainty and frustration” described that staff lacked knowledge, guidelines or faced difficult dilemmas regarding SRHR. The most difficult discussions related to intimate partner-relationship and parenthood. The staff also described that individuals with ID seldom get adapted information and are inhibited by staff, parents or society. They described strategies they had adapted to overcome the challenges and suggested areas for improvement.

### A society that begins to understand

#### A positive progress in society

The staff agreed that sexuality and sexual identity are human rights for everybody, including individuals with ID. They were also aware that legal rights and education regarding SRHR had improved. The staff voiced that individuals with ID have the right to privacy, which can be problematic for those who live in special accommodation, since they live close to other tenants and are surrounded by staff. The staff also experienced that individuals with ID wish to live their life as anyone else and that they can be more open about their needs even regarding sexuality and partnership.

*“…..there is a little more openness, …. It's not like taboo to talk about it (sexuality). I think that too has become different.”* (group 2)

#### The staff consider themselves open-minded in SRHR issues

Participants had experience of working with SRHR. Some, for example teachers and nurses, had been involved in sexuality education, others had experience of handling SRHR situations in their daily professional contact with the young people. They saw themselves as more open-minded in comparison with colleagues, parents and other actors in society. They could see positive aspects of relationship and parenthood for individuals with ID; that parenthood is natural for any human being, but with the insight that parents with ID often need more support from family members or society.

*“There are still people who say: don't wake the sleeping bear. I think it is outdated but I have heard it many times over the past few years. Not just from parents but also from staff.* (group 2)

#### Society limits the individual

It was discussed that equal SRHR may only be in theory, and that society hinders individuals with ID from an everyday life similar to others. Some had experienced that sexuality among individuals with ID evokes negative emotions in society. They could also notice reluctance among personnel and managers to talk about this topic and that SRHR is forgotten, both among themselves and by colleagues.

*“... We do not ask and do not even raise the question, don’t give them the chance to even talk, you don’t even mention sexuality…*

*- Right, I agree with you, you don’t raise the issue ... We ask how they eat and go to the toilet, but this issue is not raised.”* (Dialogue group 4)

#### Deficiency in the structure of the organisation

The staff described lack of guidelines and manuals regarding how to address SRHR among individuals with ID. They did not always know how and from where they could be supported– even if they acknowledged some improvement in terms of continued education at the workplace. Writing guidelines would, however, be complex because of the vast variety in individuals’ needs, cognitive development and maturity. The staff described that institutions and organisations such as schools, health services, and social services have difficulties to collaborate and coordinate their support. When more than one institution/organisation is involved, a person with ID may fail to understand which staff has the responsibility for what. They also found it difficult to interact with responsible authorities at the municipality.

### A complex issue for the staff to address

#### Difficult and sometimes unmanageable work

Several informants felt uncomfortable and powerless when facing issues regarding SRHR in their work. They did not know how to address problems, for example, when someone wants a partner but has difficulties managing an intimate relationship in a good way, when an individual feels frustrated for not reaching a satisfying sexuality or when he/she wants to build a family of his/her own. The feeling of powerlessness was often associated with a suspicion that an individual with ID could be subjected to neglect or abuse and that they could do very little. They also described the difficult balancing act to let someone make his/her own decisions but at the same time protect them from suffering harm.

*“… if you have the feeling that something perhaps is ongoing, and then it comes true. The powerlessness and how can you cope with that? There is like no possibility to do anything before so .... if abuse occurs, for example, then it's like your frustration comes true…”* (group 1)

Several informants had experienced talking about SRHR. Yet, some of them admitted that they felt uncertain about these issues, but that they probably were not alone in this feeling. They expressed difficulty in teaching people with ID about SRHR and challenges in describing a good relationship. It is also difficult, and time consuming, to teach individuals with ID due to a wide variety in their current knowledge, in their physical (and general) development and in their previous experiences.

*“I feel that I really have to gear myself up for, “now we’re talking about this”. After all, I have to be neutral and not turn giddy when they tell me different things what they have done and have been through…”* (group 2)

#### Uncertainty based on level of knowledge and staffs’ options/obligations

The staff expressed difficulty in knowing what options and obligations they have when complex SRHR situations emerge around individuals with ID. Parents’ obligations versus their own could be related to sexuality education, sexual hygiene and contraception. Some of the options they found unclear related to their mandate. Are they allowed to let a boyfriend sleep over in a tenant’s room or to teach someone how to masturbate in a safe and private manner? It is also unclear whose responsibility it is to talk about SRHR. A professional may feel a responsibility but without having sufficient knowledge in the subject.

*“Then, it becomes up to us, housing assistants, to all of a sudden try to address… and we usually have no major education.”* (group 1)

The informants voiced that in spite of a national curriculum for sexuality education in special schools, there is scarcity in adapted education and information materials and tools directed towards individuals with ID. Staff commented on a significant lack of knowledge among individuals with ID themselves regarding SRHR. The informants also had the experience that health services were not always capable to meet the needs of young people with ID.

*“...this with youth clinics ... if they (midwives) just stand and talk or if they also use pictures. Because it's something that I would wish that they did. The students I have, they hide their lack of knowledge. They say yes, yes... and then they leave with a lot of questions…”* (group 2)

#### Relationship and contraception – a topic to address when it appears

The staff described that many individuals with ID long for a partner. But as staff, it is very difficult to help them. They can support and encourage the person, but the disability often makes it difficult to approach other people and to create or maintain relationships. If individuals with ID are in a relationship, staff and institutions/organisations do not always support them enough to see their partner regularly or to live together if they wish.

A common example of insecurity is related to contraception. The subject is touched upon in school, but usually the staff talk about it only if they sense an individual need. The initiative to discuss contraception often comes from parents and from staff, more rarely from the individuals themselves, especially if he/she has a more severe form of ID, or a combination with other diagnoses. Staff tend to hand over the responsibility to talk about contraception to the school nurse or to the midwife at the youth clinic.

*“- I think it is mostly the parents (who propose contraception), I feel. As long as they have that power over their children, even if they are adults.*

*- Mm (several).*

*- That’s probably how it has been.*

*- I don’t think I have ever experienced that it’s a client who wants it. It is almost “Mom says”, usually it is so.”* (Dialogue group 1)

Participants described that many women with ID use contraception in order to reduce menstrual bleeding, but considered that pregnancy-prevention should also be mentioned. If this dual effect became more discussed, individuals with ID could have a chance to make their own decisions regarding contraception. According to the staff, contraceptive implants or injections are the most commonly used methods among individuals with ID. The main reason is difficulty in handling tablets, but it also makes things easier for the staff since they do not need to be involved with these long-term acting methods.

#### Reproduction and parenthood are most difficult to address

The participants described that individuals with ID think and reason about having children, both as something positive and something difficult. The staff considered it especially complicated to talk about reproduction and parenting and many were uncertain of how to discuss those topics. If a woman with ID gets pregnant, it could be viewed as a failure of the staff. However, some informants expressed that things have improved over the last years with more security for the child and better support for these families.

*“It was a man who raised the question. If he could become a father, would he and his potential partner be allowed to keep the baby?*

*- They carry those thoughts within themselves. It was clear that he felt worried. He was no more than a little over twenty years. He was fully aware of it. He wanted children, he wanted to have a family.*

*- It’s difficult to have those conversations.*

*- Yes, it’s difficult.”* (Dialogue group 2)

The staff pointed out that individuals with ID might be capable of having children, but parenthood evoked mixed feelings among them. They were afraid that a child would be mistreated or face difficulties when his/her cognitive capacity develops to a higher level than the one of his/her parents. Parenthood in general comes with challenges, and these can be especially hard for individuals with ID. However, the staff also acknowledged that parenthood gives strength and personal development. Examples of success were often related to a functioning social network of either family members or social workers. The staff discussed that parents with ID are more controlled and supervised than other parents, for example by maternal and child health services and by social services, which has both positive and negative aspects. The positive aspects include access to extended support, whereas the negative aspects could include feeling stigmatised as not being capable to manage parenthood on their own.

*“Yes, many can manage it anyway. Being positive is not the best word to describe it, but it is just mostly natural and simple. And many people also get great support from relatives and can therefore manage parenthood that way. There could be a mother with disability and the father could manage it very well and then it is like any other family.”* (group 2)

The staff found it very difficult to support individuals who know that they never will be able to take care of a child. They had no tools to address this topic and lacked knowledge. Individuals with ID may ignore what a child needs, and the staff did not know how to address these issues.

#### Social norms and own preconceptions affect the staff’s work and approach

Preconceptions affected the staff’s daily work and how they dealt with situations around individuals with ID. Some described difficulties in coping with other forms of sexuality than heterosexuality, while others described that they accepted all types of sexuality and tried to work from that perspective. However, they were aware that they inevitably were affected by a heteronormative culture, and some informants stressed the importance of making the education “non-heteronormative”. Others admitted that they educated girls more often than boys.

*“So, it’s often the girls you are targeting, both when it comes to sex talk and contraceptives and it will end up on them, that they are supposed to protect themselves.”* (group 3)

The staff testified that many individuals with ID have very engaged parents, which can be positive in many aspects. However, when parents are very close to their children, issues and situations regarding sexuality can become complex. Many individuals with ID do not want to be open about their sexuality in front of their parents. Neither do they want to present a partner to them. These circumstances were seen as problematic by some staff, while others could address it without hesitation.

*“- Then they see the one they are in love with. Yes, and you must not show outside. It's really a difficult problem, you (the staff) have the double knowledge. You find this out in confidence and then you are still supposed to sit down with these parents who may be guardians and ... and this part of life you may not mention in any way.*

*- The only thing I try to think of is that I have no obligation to tell these parents things unless the client him/herself wants it.”* (Dialogue group 1)

Staff considered that cultural differences among individuals with ID specifically affect SRHR. These differences in culture or religion are often difficult to deal with and they do not have written guidelines, translated information/education materials or specific protocols to use. Some of them reflected on how they would react if, for example, the school would teach their own children something that they, as a parent, disapproved of.

*“I have thought a lot ... If I didn't want my children to be educated, for example, in a religious congregation/school ... I almost think she should not have this (sex education). Perhaps, the parents themselves should teach their daughter? It is really difficult this, because I know it's in the curriculum.”* (group 4)

### Various strategies to work with SRHR

#### Strategies to enhance good SRHR for individuals with ID

The staff talked about the importance of respecting individuals with ID in all situations and not least regarding SRHR. It could be important to encourage individuals with ID to talk to each other. The staff can strengthen the individuals’ self-esteem and confidence and enable them to distinguish good situations from bad and thus support their decision-making ability. The informants stressed the importance of having the courage to act, but also to wait, in different situations, a judgement that could be very difficult.

#### Strategies to provide individuals with ID with knowledge about SRHR

The staff wished that individuals with ID could get information about SRHR from early age and then frequently repeated both in school and after finishing school. They stressed the importance of adequate information at a time when a person with ID is mature enough to discuss the topic, even if the information needs to be repeated many years after having left school.

*“They get sexual education in the special school, but it’s not certain that they are at that level when that education is given. Many may need to have it repeated when they are older. When you, like, have come to that stage, in the development ... and there it fails, I believe ... pretty much.”* (group 1)

The staff found it important to use adapted and positive information, not only in the housing environment but also in public institutions, for example, healthcare and social services. Examples of such information could be adapted text, words, films and other more advanced methods like the “Real care baby” simulator; looking and acting like a newborn baby. “Scare propaganda” – which was common some years ago – was not seen as useful when addressing SRHR. Participants considered it more effective to provide individuals with ID with practical knowledge and skills and to use easy everyday language in all kinds of information and give practical examples. They also voiced the importance of giving straight-forward information about parenthood rather than focussing on problems than can arise.

*“It is a human right to receive information at the level of one’s understanding.”* (group 4)

#### Strategies to achieve a good working environment

The informants discussed that support from colleagues is important to manage daily work and master difficult situations. They also felt that supervision and cooperation between professional groups, institutions and agencies are necessary to achieve a good working environment and a safe situation for individuals with ID. Many informants wished for more supervision and collaboration in order to develop and reach a higher level of competence. Guidance from other professionals was considered inadequate and thus in need of improvement. Mandates and guidelines are key strategic tools, especially the mandate from managers to raise SRHR-issues. Informants considered it desirable to work on the basis of guidelines, however, no such guidelines exist at the moment.

*“It is very important that you have the prerequisite and that you as a staff have a mandate to talk about this (SRHR issues). It should be something that a manager should say: this is what we should raise.”* (group 4)

## Discussion

The overarching theme revealed positive aspects regarding SRHR related issues for young people with ID such as a growing acceptance among the staff themselves and in society as a whole. This is a promising result and in line with other studies [18, 19] indicating that the common rights stipulated by the UN (2006) are increasingly respected. However, the theme also points towards remaining challenges such as frustration, uncertainty and stigma, which previous studies also have shown [20, 21]. The participants found discussions about having a partner and becoming a parent as the most challenging. Other studies have also highlighted this dilemma of accepting sexuality and sexual expressions among individuals with ID, but being hesitant about childbearing and parenthood [22–24].

In the absence of guidelines and appropriate tools, the staff described that they had developed various strategies, such as showing respect, supporting self-esteem and decision-making and balancing between waiting and acting when dealing with difficult situations. Ensuring privacy and respecting a person’s autonomy while at the same time protecting them from harm was another balance described by the participants. This dilemma was also described by midwives in relation to contraceptive counselling for women with ID [13, 25]. In addition to their own strategies, the participants had several suggestions for improvement such as a clear mandate, improved interprofessional collaboration and supporting policies and guidelines.

The staff voiced that everybody should be aware of their rights and have the possibility to make their own choices in life. To be able to do this, others need to contribute with education and support. Families and friends play an important role. Studies among parents to children with ID show that they often find it difficult to talk about SRHR with their children [5, 26–29] and may be reluctant to accept their sexual and reproductive choices [6, 11, 30].

In addition to families and friends, other people, such as this study population, have a key role in the life of an individual with ID. In their living environment and in the education system, children and young people with ID are surrounded by different categories of staff – represented by the participants in this study. It became clear that hey had different levels of education, training and experience. Some were not sufficiently equipped to deal with the situations they faced. There is thus a need for further training. Even though the informants acknowledged their own importance and the contribution they could have, they sometimes found it hard to distinguish their own role from that of the parents.

Age- and development-appropriate sexuality education was discussed. Even though sexuality education is mandatory in the Swedish school system, evaluations have shown that the quality sometimes is sub-standard [31]. The staff suggested more interactive and hands-on education with adapted tools, in line with a recently published pilot study [32], where an adapted toolkit “Children – what does it involve?” and a Real Care Baby simulator were used. The students who participated in an intervention with the Toolkit and the RCB simulator reported that the intervention promoted their decision-making ability in relation to future parenthood [33].

For the staff to be able to fulfil their supporting role, they need a clear mandate from managers and appropriate organisational guidelines and user-friendly pathways to follow, but also proper education and support from peers, supervisors and other professionals. Finally, society should contribute with laws, regulations and policies related to individuals with ID to facilitate a standardised approach for all institutions and organisations. The staffs were aware of the Swedish Law on Support and Service for people with ID [2] but they requested more specific guidelines related to SRHR.

### Strengths and limitations

As far as we know, this is this the first study of this type in Sweden and several important issues were raised. We believe the findings can inform the development of policy and support implementing existing policy for young people with ID. Another strength was that it included different categories of staff with a vast experience of working with young people with ID. The rules for conducting focus group interviews and analysing the data were carefully followed. We described the process in detail, so the reader can follow the analytical trail. The categories are presented with illuminating quotes. Three researchers collaborated in data collection and analysis, and the findings are in line with other studies. The discussions in the groups were vivid and everybody had the chance to contribute. The moderator used cards with key topics to guide the interviews, but often the participants touched on most topics even without the cards. Contraception did not stimulate the same vivid discussion, which may reflect the fact that few health personnel participated. This can be seen as a limitation. Another limitation is that staff talked about young people with ID, so the information about them is mirrored through the eyes of the staff. This is important to bear in mind, and the current study therefore needs to be followed by studies among young individuals with ID themselves. Further studies should focus on the situation for staff who care for young people with severe cognitive disability since they also have needs related to SRHR. Another limitation could be that the participants were recruited from a rather small geographical area and they may also have been more open-minded than staff in general, as they remarked themselves, and therefore more willing to participate in the study. Findings from qualitative studies are however not meant to be generalised, but we do believe that the findings could be transferred to similar settings as the Swedish society.

## Conclusion

Participants in the study were generally open-minded and accepting towards sexuality among young people with ID. They found it difficult to deal with reproduction/parenthood and felt unprepared and frustrated in certain situations such as the balance between respecting a person’s autonomy and privacy while protecting them from harm. The participants requested a clear mandate from managers, organizational guidelines, more education and inter-professional support. We believe these findings can inform the development of policy and support the implementation of SRHR related guidelines to support staff working with young people with ID.

## Data Availability

Not applicable due to the type of this study.

## References

[1] United Nations (2006). Convention on the rights of persons with disabilities and optional protocol, (91–86910–25-6).

[2] https://www.riksdagen.se/sv/dokument-lagar/dokument/svensk-forfattningssamling/lag-1993387-om-stod-och-service-till-vissa_sfs-1993-387 In Swedish: /Lag (1993:387) om stöd och service till vissa funktionshindrade/. Retrieved 20200502.

[3] https://www. https://nordicwelfare.org/wp-content/uploads/2017/10/Diskrimineringsskydd20och20funktionshinder20-20en20nordisk20c3b6verblick.pdf /In Swedish:/Diskrimineringsskydd och funktionhinder - en överblick/Protection against discrimination and functional impairment - an overview/. Retrieved 20200502.

[4] Rushbrooke E, Murray CD, Townsend S (2014). What difficulties are experienced by caregivers in relation to the sexuality of people with intellectual disabilities? A qualitative meta-synthesis. Res Dev Disabil.

[5] Eastgate G, Scheermeyer E, van Driel ML, Lennox N (2012). Intellectual disability, sexuality and sexual abuse prevention - a study of family members and support workers. Aust Fam Physician.

[6] Evans DS, BE MG, Healy E, Carley SN (2009). Sexuality and personal relationships for people with an intellectual disability. Part II: staff and family carer perspectives. J Intellect Disabil Res.

[7] Ledger S, Earle S, Tilley E, Walmsley J (2016). Contraceptive decision-making and women with learning disabilities. Sexualities.

[8] van Schrojenstein Lantman-de Valk HM, Rook F, Maaskant MA (2011). The use of contraception by women with intellectual disabilities. J Intellect Disabil Res.

[9] McCarthy M (2009). Contraception and women with intellectual disabilities. J Appl Res Intellect Disabil.

[10] Greenwood NW, Wilkinson J (2013). Sexual and reproductive health care for women with intellectual disabilities: a primary care perspective. Int J Family Med.

[11] Cuskelly M, Bryde R (2004). Attitudes towards the sexuality of adults with an intellectual disability: parents, support staff, and a community sample. J Intellect Develop Disabil.

[12] Höglund B, Lindgren P, Larsson M (2013). Midwives’ knowledge of, attitudes towards and experiences of caring for women with intellectual disability during pregnancy and childbirth: a cross-sectional study in Sweden. Midwifery.

[13] Höglund B, Larsson M (2019). Midwives’ work with and attitudes towards contraceptive counselling and contraception among women with intellectual disability– focus group interviews in Sweden. Eur J Contracept Reprod Health Care.

[14] Löfgren-Mårtenson L (2008). Love in cyberspace: Swedish young people with intellectual disabilities and the internet. Scand J Disabil Res.

[15] Elo S, Kyngas H (2008). The qualitative content analysis process. J Adv Nurs.

[16] Hsieh HF, Shannon SE (2005). Three approaches to qualitative content analysis. Qual Health Res.

[17] Graneheim UH, Lundman B. Qualitative content analysis in nursing research: concepts, procedures and measures to achieve trustworthiness. Nurse Educ Today. 2004, 24;(2):105–12. 10.1016/j.nedt.2003.10.001. 10.1016/j.nedt.2003.10.001 14769454

[18] MacLachlan M, Mannan H, Huss T, Munthali A, Amin M (2016). Policies and processes for social inclusion: using EquiFrame and EquIPP for policy dialogue. Comment on “are sexual and reproductive health policies designed for all? Vulnerable groups in policy documents of four European countries and their involvement in policy development”. Int J Health Policy Manag.

[19] Yeager P, Kaye HS, Reed M, Doe TM (2006). Assistive technology and employment: experiences of Califonians with disabilities. Work..

[20] Stenfert Kroese B, Rose J, Heer K, O’Brien A (2013). Mental health Services for Adults with intellectual disabilities - what do Service users and staff think of them?. J Appl Res Intellect Disabil.

[21] Pelleboer-Gunnink HA, Van Oorsouw WMW, Van Weeghel J, Embregts PJCM (2017). Mainstream health professionals’ stigmatising attitudes towards people with intellectual disabilities: a systematic review. J Intellect Disabil Res.

[22] Theodore K, Foulds D, Wilshaw P, Colborne A, Nga Yu Lee J, Mallaghan L (2018). We want to be parents like everybody else’: stories of parents with learning disabilities. Int J Dev Disabil.

[23] Feldman MA, McConnell D, Aunos M (2012). Parental cognitive impairment, mental health and child outcomes in a child protection population. J Ment Health Res Intellect Disabil.

[24] Mayes R, Llewellyn G. Mothering differently: narratives of mothers with intellectual disability whose children have been compulsorily removed. J Intellect Develop Disabil. 37(2):121–30. 10.3109/13668250.2012.673574. 10.3109/13668250.2012.673574 22545776

[25] Höglund B, Larsson M (2019). Ethical dilemmas and legal aspects in contraceptive counselling for women with intellectual disability - focus group interviews among midwives in Sweden. J Appl Res Intellect Disabil.

[26] Gürol A, Polat S, Oran T (2014). Views of mothers having children with intellectual disability regarding sexual education: a qualitative study. Sex Disabil.

[27] Lafferty A, McConkey R, Simpson A (2012). Reducing the barriers to relationships and sexuality education for persons with intellectual disabilities. J Intellect Disabil.

[28] Pownell JD, Jahoda A, Hastings R, Kerr L (2011). Sexual understanding and development of young people with intellectual disabilities: mothers’ perspectives of within-family context. Am J Intellect Dev Disabil.

[29] Isler A, Tas F, Beytut D, Conk Z (2009). Sexuality in adolescents with intellectual disabilities. Sex Disabil.

[30] Löfgren-Mårtenson L (2004). “May I?” about sexuality and love in the new generation with intellectual disabilities. Sex Disabil.

[31] Skolinspektionen. Sex och samlevnadsundervisning. /In English: Sexuality education. Retrived from: https://www.skolinspektionen.se/globalassets/publikationssok/granskningsrapporter/kvalitetsgranskningar/2018/sex-och-samlevnad/sex-och-samlevnadsundervisning-rapport-feb-2018.pdf.

[32] Janeslätt G, Larsson M, Wickström M, Springer L, Höglund B (2018). An intervention using the parenting toolkit “children-what does it involve?” and the real-care-baby simulator among students with intellectual disability-a feasibility study. J Appl Res Intellect Disabil.

[33] Randell E, Janeslätt G, Höglund B (2020). A school-based intervention promoting insights in future parenting among students with intellectual disability – a Swedish interview study.

